# Fe(III)-dependent Nrf activity determines nitrate reduction partitioning in nitrate-reducing communities

**DOI:** 10.1128/mbio.02220-25

**Published:** 2025-09-30

**Authors:** Ji Zhan, Lu Zhang, Shuyao Lai, Junhui Guo, Tianqi Lin, Guohong Liu, Christopher Rensing, Xing Liu, Shungui Zhou

**Affiliations:** 1 Fujian Provincial Key Laboratory of Soil Environmental Health and Regulation, College of Resources and Environment, Fujian Agriculture and Forestry University 12449, Fuzhou, China; 2 Institute of Resources, Environment and Soil Fertilizer, Fujian Academy of Agricultural Sciences 107629, Fuzhou, Fujian, China; The University of Oklahoma, Norman, Oklahoma, USA

**Keywords:** dissimilatory nitrate reduction to ammonium, denitrification, interspecies synergistic denitrification, nitrite reductase, nitrogen conversion, Fe(III) cofactor

## Abstract

Identifying the factors that affect the nitrate reduction partitioning between dissimilatory nitrate reduction to ammonium (DNRA) and denitrification is crucial for mitigating nitrogen loss in ecosystems. Conventionally, the nutrient status of the environment (e.g., the carbon-to-nitrogen ratio) is recognized as the key determinant of nitrogen conversion pathways. Here, we report that the availability of Fe(III) regulates the nitrate reduction partitioning in *Geobacter metallireducens* and *Alcaligenes faecalis* co-culture. We controlled the availability of Fe(III) in the coculture medium and tracked nitrogen conversion dynamics and community composition. The results demonstrated that the coculture performed DNRA, contributed mainly by *G. metallireducens* under Fe(III)-replete conditions, while performing interspecies synergistic denitrification between both species under Fe(III)-depleted conditions. Nitrate/nitrite reductase activity calculations and mutation analyses indicated that nitrate reduction partitioning in the coculture was governed by the nitrite reductase (Nrf) activity of *G. metallireducens*, which was Fe(III)-dependent. Further validation in urban river water confirmed that Fe(III) supplementation significantly enhances DNRA activity. Our findings establish Fe(III) as a previously unrecognized regulator of microbial nitrogen retention, showing insights into strategies for managing nitrogen fluxes in agricultural and aquatic systems.

**IMPORTANCE:**

Nitrogen is essential for life, but its loss from ecosystems through microbial processes like denitrification harms agricultural productivity and contributes to greenhouse gas emissions. Retaining nitrogen as ammonium via microbial dissimilatory nitrate reduction to ammonium (DNRA) could mitigate these issues, but the factors governing microbial prioritization of DNRA over denitrification remain unclear. Our study reveals that Fe(III) plays a critical, previously unrecognized role in steering this process. We show that Fe(III) availability determines whether the nitrate-reducing community conserves nitrogen as ammonium or releases it as gas, with implications for managing nitrogen in soils and waterways. By demonstrating Fe(III)’s ability to enhance nitrogen retention in environmental systems like urban rivers, our findings offer a new lever for sustainable agriculture and pollution control. This work bridges microbial ecology and environmental management, highlighting how trace metals shape nutrient cycles in ways that can be harnessed to protect ecosystem health.

## INTRODUCTION

Nitrate is a key nutrient for plant growth and is essential for ensuring food productivity to support the development of human civilization (1, 2). It has been estimated that agricultural fertilization accounts for >12.92 million tons of nitrate annually (3). However, nitrogen fertilizer is only partially used by plants and inevitably dissipates after fertilization (4). Microbial reduction is the main nitrate dissipation pathway (>50%) (5). There are two microbial-contributed nitrate reduction: dissimilatory nitrate reduction to ammonium (DNRA) and denitrification (6, 7). In DNRA, nitrate is first reduced to nitrite and subsequently reduced to ammonium. The generated ammonium has been shown to either be easily assimilated by plants or preserved in soil due to electrostatic force interactions between positively charged ammonium and negatively charged soil particles (8). Therefore, nitrogen is conserved after DNRA. In contrast, in denitrification, nitrate is converted to gaseous nitrogen species, nitrogen is lost, and the greenhouse gas nitrous oxide is occasionally produced (9). Generally, these two distinct nitrate reduction partitioning processes are performed by different microorganisms and can coexist in natural environments (10, 11). Thus, inducing nitrate reduction partitioning toward DNRA is favorable for conserving nitrogen sources from ecosystems.

Nitrate reduction is usually a respiratory process of microorganisms in oxygen-limited environments (12, 13). Therefore, it is generally recognized that microbial nitrate reduction partitioning is affected by the nutrient status of the environment. For example, the C/NO_3_^−^ ratio has been identified as the primary factor. An elevated C/NO_3_^−^ ratio usually leads to DNRA dominance in anoxic soils and sediments due to different nitrate affinities between nitrate ammonifiers and denitrifiers (6, 14–16). A high nitrite/nitrate ratio also selects DNRA in coastal sediments due to a higher energy and more metabolic electrons being generated during DNRA (14, 17, 18). Additionally, the abundance of sulfide favors DNRA in salt marsh sediments by providing a competitive edge to nitrate ammonifiers while inhibiting denitrifiers (19, 20). Fe(II) has been shown to power the DNRA while inhibiting denitrification in estuary sediments and river floodplains (21, 22). When Fe(II) is provided, the nitrate ammonifier oxidizes Fe(II) for DNRA; therefore, DNRA dominates. However, when Fe(II) is depleted, denitrification dominates again (13, 23, 24). Other environmental factors, such as pH and redox status, have also been suggested (18, 25). To be specific, DNRA is usually preferred in alkaline environments or under conditions with high redox potentials. Notably, all of these factors act to shape the nitrate reduction community to affect nitrate reduction partitioning.

Microbial nitrate reduction is performed by nitrate reductase (Nar and Nap) and nitrite reductase (Nrf, Nir, and ONR) (26, 27). The latter is a metalloenzyme that catalyzes the microbial nitrite reduction and determines the nitrate reduction to ammonium or gaseous nitrogen (26). Specifically, Nrf is an iron-based cytochrome *c* and is able to convert nitrite to ammonium (28, 29). Nitrite reductase of NirK and NirS is iron-based or copper-containing and has been shown to reduce nitrite to nitric oxide (28). The expression of nitrite reductase is under cellular regulation and affected by the living environment (29, 30). Therefore, some of the aforementioned environmental factors have also been shown to regulate the expression of a specific nitrite reductase to modulate the nitrate reduction partitioning (30). In the meantime, the activity of the enzyme determines the catalytic kinetics and can affect the catalytic reaction. The enzyme activity is generally correlated with reaction conditions. Although a cell provides a relatively stable environment for intracellular enzymatic reactions, the activity of an enzyme can still be affected by various environmental factors (31, 32). Thus, we speculated that some environmental factors could tune the activity of nitrite reductase to modulate nitrate reduction partitioning in a nitrate-reducing community.

In this study, we first studied the factor that affects nitrate reduction partitioning in a nitrate-reducing coculture consisting of a nitrate ammonifier (*Geobacter metallireducens*) and a denitrifier (*Alcaligenes faecalis*). The results demonstrate that nitrate reduction partitioning of the coculture can be modulated by Fe(III). Specifically, the coculture performs DNRA under ferruginous conditions, whereas it turns to denitrification in a non-ferruginous environment because Fe(III) can tune the Nrf activity of *G. metallireducens*, which is Fe(III)-dependent. Additionally, we show that Fe(III) supplementation promotes nitrate ammonification during nitrate reduction in urban river water. Our study provides a better understanding of nitrate reduction partitioning in nitrate reduction environments and nitrogen retention in aquatic ecosystems.

## MATERIALS AND METHODS

### Microbial strains and culture conditions

*G. metallireducens* strain GS15 (ATCC 53774) and *A. faecalis* (DSM 30030) were purchased from the American Type Culture Collection and China General Microbiological Culture Collection Center (CGMCC), respectively. *G. metallireducens* strains were routinely cultivated in an Fe(III) abundant medium (FCA medium), as previously described (33), or grown in nitrate medium (34). *A. faecalis* was cultured in a Fe(III)-deficient coculture medium (FWNN medium) (35, 36) supplemented with 15 mmol·L^−1^ sodium acetate and 5 mmol·L^−1^ sodium nitrite as the electron donor and acceptor, respectively. To grow the coculture, the coculture medium was supplemented with 15 mmol·L^−1^ sodium acetate and 5 mmol·L^−1^ sodium nitrate. Soluble ferric citrate was added as the Fe(III) source as needed (37). All cultures were anaerobically cultured with Ar/CO_2_ (80:20) as headspace gas at 30°C.

### Nitrogen conversion characterization

The nitrate, nitrite, and ammonium concentrations were determined using dual-wavelength ultraviolet spectrophotometry, N-(1-naphthyl)-ethylenediamine hydrochloride colorimetry, and indophenol blue colorimetry, respectively, with a UV-vis spectrophotometer (Shimadzu UV-2600, Japan), as previously reported (38, 39). The N_2_O and N_2_ in the gaseous phase were measured using a gas chromatograph (GC, Agilent Technologies 7890B, USA) equipped with a peristaltic pump (Gilson Miniplus 3, Gilson, France) and two detectors (an electron capture detector and a thermal conductivity detector). To ensure that the air pressure was balanced in the anaerobic bottle, a fixed volume of sterile Ar/CO_2_ (80:20) mixture was injected into the anaerobic bottle after each sampling. The N_2_O and N_2_ concentrations were calculated relative to the liquid volume. All tests were performed in triplicates.

### Cell and gene quantification

The abundances of *G. metallireducens* and *A. faecalis* in the coculture were quantified via quantitative polymerase chain reaction (qPCR), which was conducted using the LightCycle 96 System (Roche Applied Science, Penzberg, Germany) with iTaq Universal SYBR GREEN Supermix (Bio-Rad, USA). The cells were collected by centrifugation (8,000 × *g*, 10 min), and the genomic DNA was extracted using a DNeasy PowerBiofilm Kit (QIAGEN, Germany). To construct the standard curves, two plasmids, pMD19-Gm and pMD19-Af, were first constructed. In detail, the primer pair GmetFor/GmetRev was used to amplify a unique sequence (Gmet_2143) with the genome of *G. metallireducens* as a template and subsequently cloned into the plasmid pMD19-T (Takara Biomedical Technology, Beijing, China) to generate the plasmid pMD19-Gm. The primer pair AfF/AfR was used to amplify a unique sequence (CPY64_00555-CPY64_00560) from the genome of *A. faecalis* to generate the plasmid pMD19-Af after cloning into the plasmid pMD19-T. The two plasmids were 10-fold serially diluted and then used as templates to make standard curves. To quantify *G. metallireducens* and *A. faecalis* cells, the primer pairs qGmet_F/qGmet_R and qAfF/qAfR were used, respectively, with the genomic DNA of the coculture as template. Therefore, the number of each species in the coculture could be calculated by counting the species-specific gene copy numbers after calculating against the standard curves. Similarly, qPCR was conducted to quantify the abundance of the *nirK*, *nirS,* and *nrfA* genes in urban river water nitrate-reducing communities, and the primer pairs F1aCu/R3Cu, Cd3aF/R3cd, and nrfAF2aw/nrfAR1 were used for standard curve construction and gene quantification (40, 41). All primers used in this study are listed in Table S1. The experiment details and standard curve calculations are specified in Table S2.

### Mutant construction

All bacterial strains and plasmids in this study are listed in Table S3. *G. metallireducens* mutants were constructed following the reported methods (42, 43). Two nominated nitrate reductase genes were knocked out: *narG1* and *narG2* (GenBank locus Gmet_0329 and Gmet_1020, respectively). In brief, three DNA fragments were prepared: the upstream (500 bp) and downstream (500 bp) sequences of *narG1* were amplified by primer pairs narG1upFor/narG1upRev and narG1dnFor/narG1dnRev, respectively, with *G. metallireducens* genomic DNA as a template; the primer pair spef/sper was used to amplify the spectinomycin resistance cassette flanked by the *loxP* sequence using the plasmid pRG5 as a template (44). These three fragments were connected with the linear plasmid pUC19 using an In-Fusion HD Cloning Kit (Takara Biomedical Technology, Beijing, China), which generated the plasmid pUC-GMnarG1. Similarly, two DNA fragments were prepared: the 500 bp upstream and 500 bp downstream sequences of *narG2* were amplified by the primer pairs of narG2upFor/narG2upRev and narG2dnFor/narG2dnRev, respectively. These two fragments were combined with the spectinomycin resistance cassette and connected with pUC19 to generate the plasmid pUC-GMnarG2. To mutate *narG1*, the plasmid pUC-GMnarG1 was linearized with the NcoI enzyme (New England BioLabs (NEB), Ipswich, MA, USA) and electroporated into electrocompetent *G. metallireducens*, which generated the strain G.m-*ΔnarG1^Sp^*. Thereafter, the pCM158 plasmid was transferred into the G.m-*ΔnarG1^Sp^* strain to induce the excision of the spectinomycin resistance cassette and generate the G.m-*ΔnarG1* strain. To further mutate *narG2*, the linearized plasmid pUC-GMnarG2 was electroporated into G.m-*ΔnarG1*. A double-gene mutant of G.m-*ΔnarG* was generated. The *G. metallireducens* strain deficient in the nitrite reductase NrfAH (GenBank loci Gmet_0294 to Gmet_0296) was constructed via the same method and was named G.m-*ΔnrfA*. All mutants were confirmed via PCR (Fig. S1).

### Nitrate and nitrite reductase activity measurement

*G. metallireducens* was first grown in coculture medium supplemented with different Fe(III) citrate concentrations. The cells were collected at the late logarithmic phase and lysed with an ultrasonic homogenizer (JY92-IIDN, Ningbo Scientz Biotechnology Co., Ningbo, China). The protein concentrations of the cell lysate were measured using a microBCA protein assay (Micro BCA protein assay kit, Thermo Fisher Scientific, Rockford, USA) following the manufacturer’s instructions. Nitrate reductase and nitrite reductase activities were determined using a Nitrate Reductase (NR) Activity Assay Kit (BC0080, Solarbio, China) and an NiR Assay Kit (BC1545, Solarbio, China) (45), respectively, and calculated against the amount of protein used.

### Transcriptome sequencing and analysis

Cell samples were quickly frozen with liquid nitrogen and stored at −80°C before use. TRIzol reagent (Invitrogen, California, USA) was used to extract the total RNA as previously described (46). Transcriptome sequencing was conducted by Magigene Technology Co., Ltd. (Guangzhou, China). Briefly, total RNA was treated with an Epicenter Ribo-Zero rRNA Removal Kit to obtain purified mRNA. The NEBNextő Ultra II Directional RNA Library Prep Kit for Illumina was used for subsequent sequencing. The entire genomes of *G. metallireducens* (NC_007517.1) and *A. faecalis* (NZ_CP023667.1) from NCBI were used as references. Fragments per kilobase per million mapped reads were adopted to normalize the map reads of each gene. Then, DESeq2 and edgeR were used to analyze the differential expression. To obtain the FDR, the *P* value was corrected using the Bonferroni method (47).

### DNA extraction and high-throughput sequencing

Genomic DNA was extracted from urban river water using the E.Z.N.A. soil DNA Kit (Omega Bio-tek, Norcross, Georgia, USA). The primer pairs 515F/806R and nrfAF2aw/nrfAR1 were utilized to amplify 16S rRNA and *nrfA* genes. The purified amplicons were paired-end sequenced on an Illumina NextSeq2000 platform (Illumina, San Diego, USA) by Majorbio Bio-Pharm Technology Co. Ltd. (Shanghai, China). Microbial communities analysis based on the OTUs information was performed on the online Majorbio Cloud platform (Majorbio Bio-Pharm Technology Co. Ltd., Shanghai, China).

### Total iron determination

A 100 µL sample was mixed with 900 µL of freshly prepared hydroxylamine hydrochloride (0.25 mol·L^−1^ in 0.5 mol·L^−1^ HCl). The mixture was incubated at 60°C for 2 h to induce all Fe(III) to be reduced to Fe(II). The Fe(II) concentration was determined with the ferrozine assay as previously reported (42).

## RESULTS AND DISCUSSION

### Nitrate reduction partitioning in *G. metallireducens* and *A. faecalis* coculture

**Fig 1:**
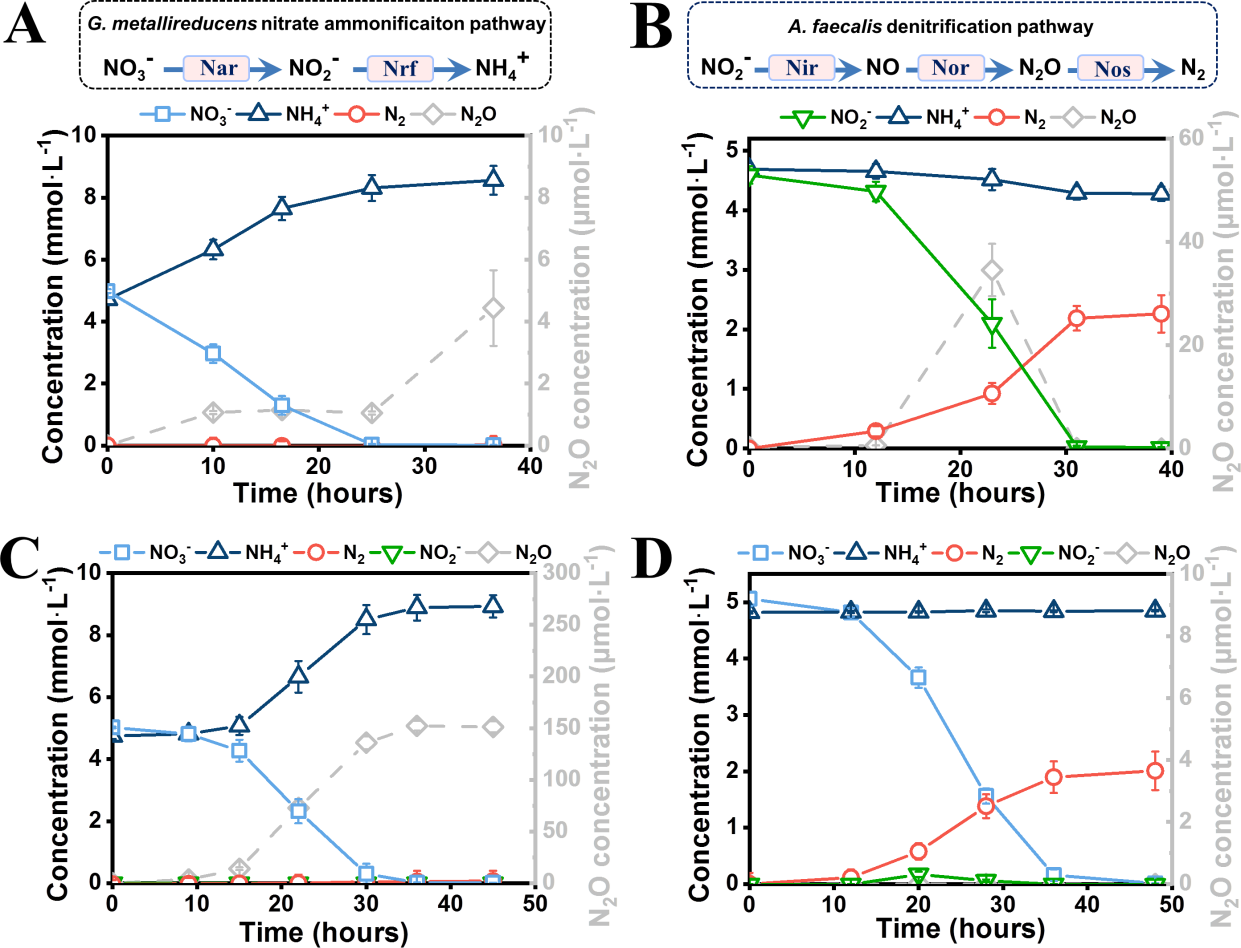
Nitrate reduction characterization. (**A**) Nitrate reduction pathway and nitrogen conversion of *G. metallireducens* during nitrate reduction. *G. metallireducens* conducted DNRA when grown in a nitrate medium. (**B**) Nitrite reduction pathway and nitrogen conversion of *A. faecalis* during nitrite reduction. *A. faecalis* performed denitrification in coculture medium. (**C**) Nitrogen conversion of the *G. metallireducens* and *A. faecalis* coculture during the primary coculturing. The coculture performed DNRA in the coculture medium. (**D**) Nitrogen conversion of the *G. metallireducens* and *A. faecalis* coculture after the primary coculture had been transferred to a fresh coculture medium. The coculture mainly conducted denitrification.

*G. metallireducens* is a typical nitrate ammonifier (48); it does not contain the nitrite reductase genes *nirK* and *nirS* (49), which encode key enzymes in the denitrification pathway that catalyze the reduction of nitrite to nitric oxide. However, the genome encodes the nitrite reductase NrfA, which catalyzes the respiratory ammonification (Fig. 1A) (50). Thus, *G. metallireducens* grows in nitrate medium and reduces nitrate to ammonium (Fig. 1A), as previously reported (48). In particular, a byproduct of nitrous oxide can also be detected (Fig. 1A) (51). In contrast, *A. faecalis* is a typical denitrifier; it harbors the entire denitrification pathway except for nitrate reductase (Fig. 1B), but it does not contain genes that encode the nitrite ammonification pathway (52). Therefore, *A. faecalis* cannot reduce nitrate (Fig. S2) and only reduces nitrite for nitrogen generation (Fig. 1B). We simultaneously inoculated (1% vol/vol inoculum) these two species into a coculture medium with nitrate as the only electron acceptor. As indicated in Fig. 1C, the coculture performed DNRA with a small amount of generated nitrous oxide. We also analyzed the coculture composition by quantifying the species-specific genes via qPCR. The results show that *G. metallireducens* dominated the nitrate ammonification coculture (ca. 99%). Therefore, nitrate reduction was mainly contributed by *G. metallireducens* in the coculture system, and ammonium was consequently generated.

We further subcultured the ammonification coculture (1% vol/vol inoculum). Surprisingly, the dominant nitrate reduction conversion became denitrification for dinitrogen gas generation (Fig. 1D). Therefore, a denitrifying coculture was formed. Additionally, coculture flocs formed (Fig. S3), and the ratio of *G. metallireducens* to *A. faecalis* changed drastically to ca. 1:1, which indicates a collaborative denitrification. In particular, nitrite accumulation was temporally detected during nitrate reduction in the coculture system, whereas no nitric oxide or nitrous oxide was detected at any point. Considering *A. faecalis* could not reduce nitrate alone in the coculture medium, the results suggest that in the new denitrifying coculture, most *G. metallireducens* must have undergone synergistic denitrification with *A. faecalis* for nitrate reduction to nitrogen gas. Notably, the synergistic relationship appears stable, since the nitrate reduction partitioning pattern and ratio of the two species in the coculture did not change after continuous subculturing (data not shown).

### The availability of Fe(III) affects nitrate reduction partitioning in the coculture

**Fig 2:**
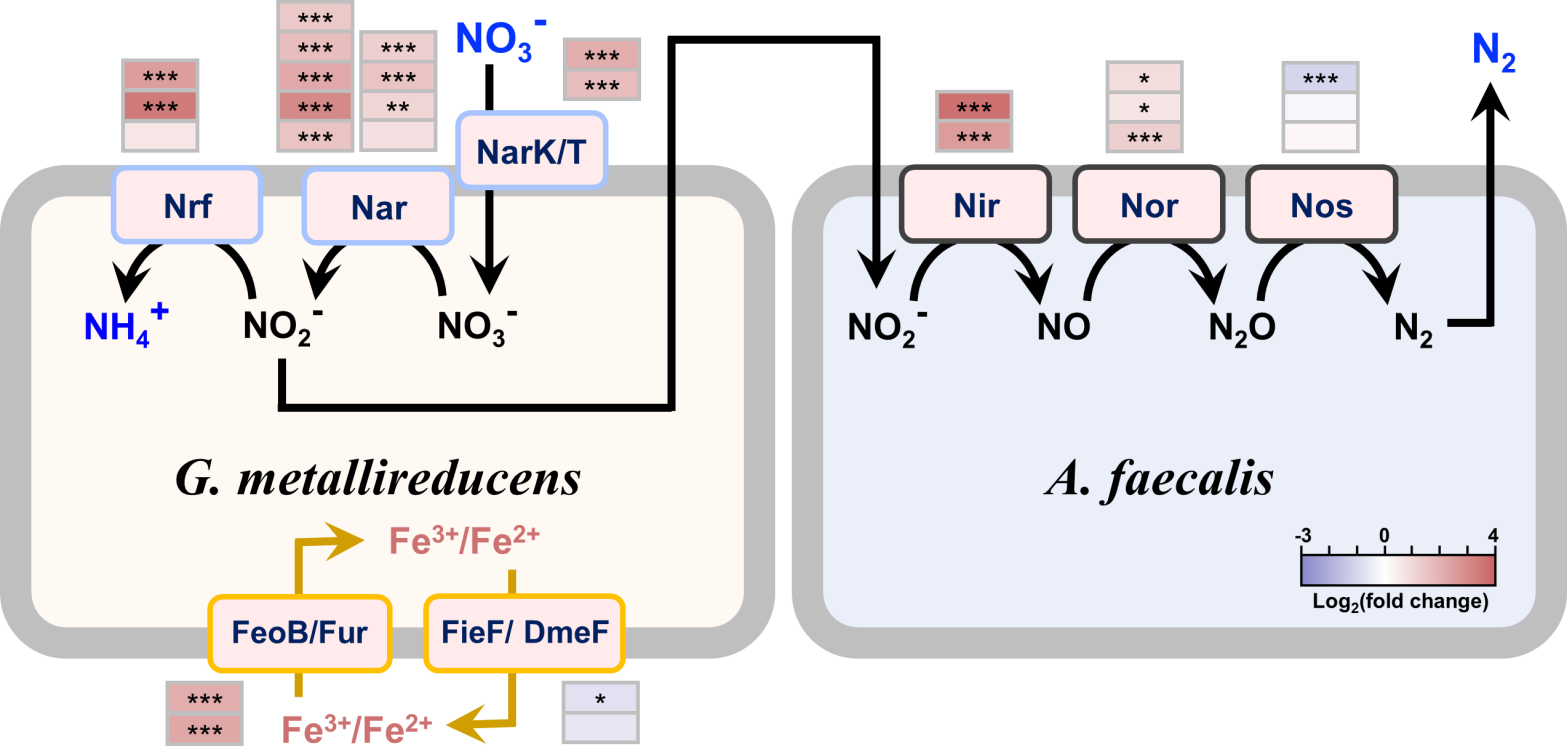
Expression of nitrate-reduction-related genes in the *G. metallireducens* and *A. faecalis* denitrification coculture. *G. metallireducens* cells that perform DNRA and *A. faecalis* cells that execute denitrification were selected as the references. Nar: nitrate reductase; Nir and Nrf: nitrite reductase; Nor: nitric oxide reductase; Nos: nitrous oxide reductases; FeoB/Fur: iron importer; FieF/DmeF: iron exporters; NarK: nitrate/nitrite transporter; NarT: nitrate/nitrite antiporter. ***P < 0.001, **P < 0.01, *P < 0.05.

We further analyzed the gene expression of each of the two species in the denitrifying coculture (Data S1 and S2). *G. metallireducens* cells that underwent nitrate ammonification and *A. faecalis* cells that reduced nitrite to nitrogen gas were cultured separately for comparison. As Fig. 2 shows, in the denitrification coculture, the genes that encoded nitrite reductase (Nir) and nitric oxide reductase (Nor) were more highly expressed, whereas the gene that encoded nitrous oxide reductase (Nos) was slightly less expressed in *A. faecalis,* which indicates the active expression of the denitrification pathway. Similarly, the genes involved in the nitrate ammonification pathway, including those that encode nitrate reductase (Nar) and nitrite reductase (Nrf), were highly expressed in *G. metallireducens*. The high expression of the nitrate ammonification pathway excludes the possibility that the suppressed expression of the DNRA pathway contributed to the denitrification coculture. In addition, the active expression of the denitrification pathway in *A. faecalis* provides further evidence of the synergistic denitrification in the coculture. Significantly, the expression of genes that encode the nitrate/nitrite transporter (NarK) and nitrate/nitrite antiporter (NarT), which facilitate the secretion of nitrite from the nitrate reduction in *G. metallireducens,* was also upregulated (53). Considering the temporary accumulation of nitrite in the coculture system, the results indicate a deficiency in nitrite reduction in *G. metallireducens* and suggest the possibility that interspecies synergistic denitrification depends on the nitrite exchange.

**Fig 3:**
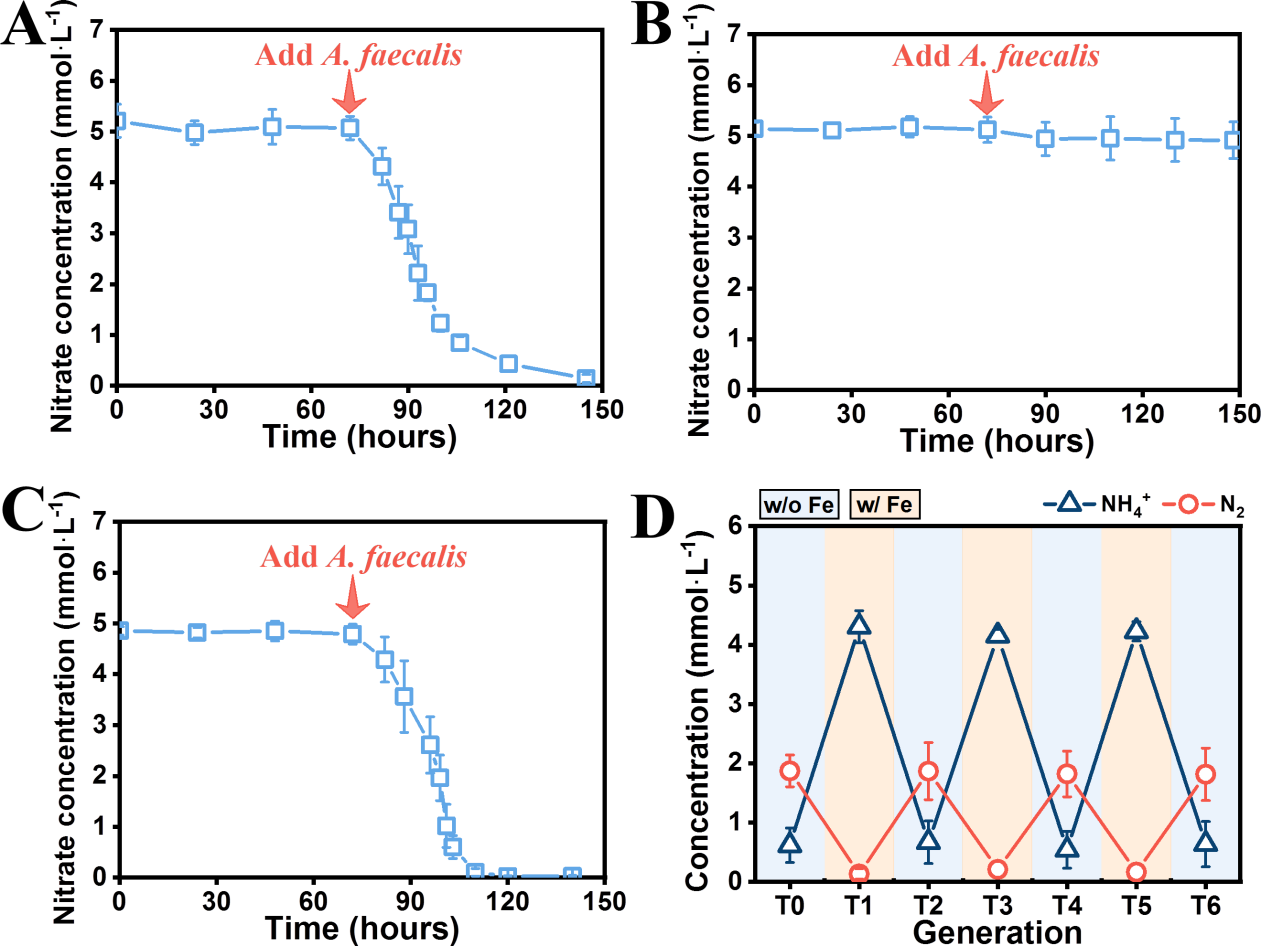
Nitrate reduction characterization. (**A**) Nitrate reduction by *G. metallireducens* in a coculture medium. *G. metallireducens* was washed twice with a coculture medium before inoculation. *G. metallireducens* was not able to reduce nitrate until the addition of *A. faecalis* to the medium. (**B**) Nitrate reduction of the *G. metallireducens* nitrate reductase-deficient strain (strain G.m-Δ*narG*) in a coculture medium. The addition of *A. faecalis* was not able to recover the nitrate reduction of the strain G.m-Δ*narG*. (**C**) Nitrate reduction of the *G. metallireducens* nitrite reductase-deficient strain (strain G.m-Δ*nrfA*) in a coculture medium. The addition of *A. faecalis* was able to recover the nitrate reduction of the G.m-Δ*nrfA strain*. (**D**) Nitrogen conversion of the *G. metallireducens* and *A. faecalis* coculture that was intermittently treated with Fe(III) during six transfers. The availability of Fe(III) modulated the nitrate reduction partitioning of the coculture between DNRA and denitrification.

The transcriptomics results of *G. metallireducens* also revealed that the expression of genes that encoded those membrane transport proteins was significantly affected after the formation of a denitrifying coculture. In particular, the iron importer encoded by *feoB/fur* was upregulated, whereas the iron exporter encoded by *fieF/dmeF* was downregulated, which indicates that the *G. metallireducens* cells might be experiencing iron deficiency when grown in the coculture medium. A previous study indicated that *G. metallireducens* only performed nitrate reduction to ammonium under iron-rich conditions (54). Since our coculture medium was iron-deficient, we speculated that even though the nitrate ammonification pathway is highly expressed, the catalytic reaction of nitrate ammonification by *G. metallireducens* might have been severely suppressed in the coculture; therefore, *G. metallireducens* underwent synergistic denitrification with *A. faecalis* to adapt to the iron deficiency for survival. To verify this speculation, we focused on limiting the Fe(III) during coculturing by inoculating the coculture medium with washed *G. metallireducens* cells to prevent the carry-over of Fe(III) from the *G. metallireducens* growth medium. Significantly, the two species formed a denitrifying coculture during the primary coculturing (Fig. S4). In addition, similar to a previous report (54), *G. metallireducens* could not reduce nitrate in the coculture medium (Fig. 3A). However, when supplemented with live *A. faecalis* rather than dead *A. faecalis*, nitrate reduction was recovered (Fig. 3A and 5). Moreover, the addition of *A. faecalis* was not able to recover the nitrate reduction of a *G. metallireducens* mutant strain that lacked nitrate reductase (Fig. 3B) but recovered the nitrate reduction of a mutant strain that lacked the gene encoding nitrite reductase (Fig. 3C). These results demonstrate interspecies synergistic denitrification in the denitrification-dominated coculture where *G. metallireducens* reduces nitrate to nitrite, which is transferred to *A. faecalis* and thereafter reduced to nitrogen by *A. faecalis*.

Since the Fe(III) deficiency can contribute to the formation of a denitrifying coculture, how does the denitrifying coculture behave under Fe(III) sufficiency? To answer this question, we added Fe(III) (0.3 mmol·L^−1^ ferric citrate) to the coculture medium to subculture the denitrifying coculture. A nitrate ammonification coculture recovered with *G. metallireducens* dominated (Fig. S6). In particular, the coculture could consistently perform nitrate ammonification with *G. metallireducens* dominated (accounting for ca. 99%) during continuous subculturing in a coculture medium supplemented with Fe(III). Notably, no significant Fe(II) accumulation was observed in the system, which rules out the possibility that Fe(III) serves as a supplemental electron acceptor for *G. metallireducens*. These results demonstrate that the availability of Fe(III) affects nitrate reduction partitioning in the coculture. Furthermore, Fe(III) could act as a stable factor to determine the nitrate reduction partitioning (DNRA with Fe(III) vs. denitrification without Fe(III)) and microbial community composition of the coculture during continuous alternate transfer between coculture medium supplemented with and without Fe(III) (Fig. 3D and Fig. S7).

Considering the presence of Fe(III) contributes to an ammonification coculture, we speculate that the formation of an ammonification coculture dominated by *G. metallireducens* during the primary coculturing (Fig. 1C) must be due to the carry-over of Fe(III) of *G. metallireducens,* which was grown in an FCA medium with Fe(III) as the electron acceptor. We measured the Fe(III) concentration of the coculture medium after the primary coculturing. As expected, a concentration of 0.26 mmol·L^−1^ Fe(III) was detected. However, after the first transfer, the Fe(III) concentration has been diluted 10,000 times, which might have been below the effective Fe(III) concentration to support nitrate reduction to ammonium by *G. metallireducens,* and thereafter a synergistic denitrifying coculture was formed (Fig. 1D). In summary, these results suggest that Fe(III) is the primary factor that controls the nitrate ammonification activity of *G. metallireducens* and subsequently determines the nitrate reduction partitioning of the *G. metallireducens* and *A. faecalis* coculture.

### Fe(III) determines the nitrite reductase (Nrf) activity of *G. metallireducens* in the coculture

**Fig 4:**
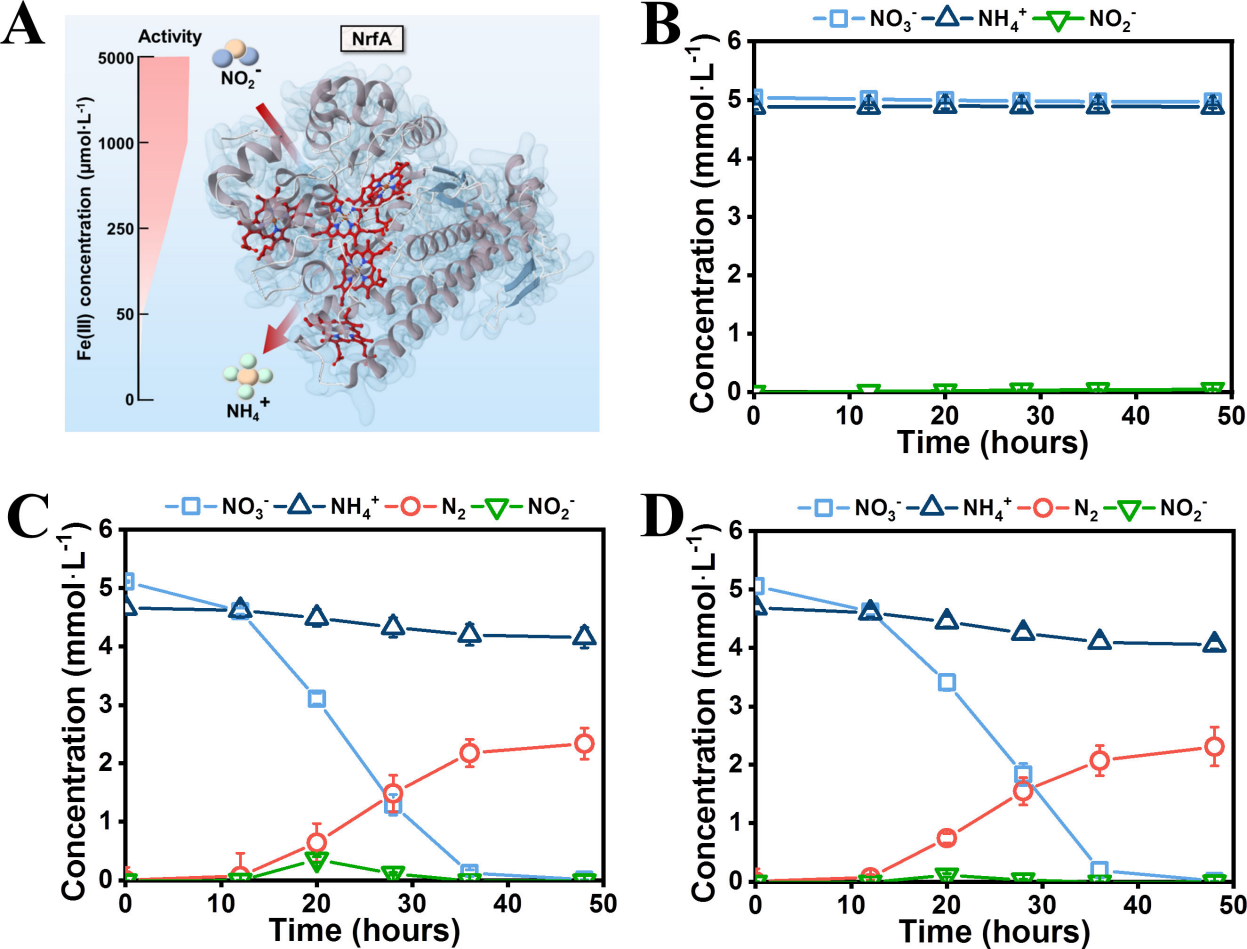
Effect of the Fe(III)-dependent Nrf activity on the nitrate reduction in the coculture. (**A**) Nrf activity of *G. metallireducens* cell lysates grown in nitrate medium supplemented with varying Fe(III) concentrations. (**B**) Nitrogen conversion of the *G. metallireducens* mutant strain G.m-Δ*nrfA* (deficient in nitrite reductase) growing in a nitrate medium. (**C**) Nitrogen conversion of the G.m-Δ*nrfA* and *A. faecalis* coculture growing in a coculture medium. (**D**) Nitrogen conversion of the G.m-Δ*nrfA* and *A. faecalis* coculture growing in a coculture medium supplemented with Fe(III). The coculture still performed denitrification in the presence of Fe(III).

**Fig 5:**
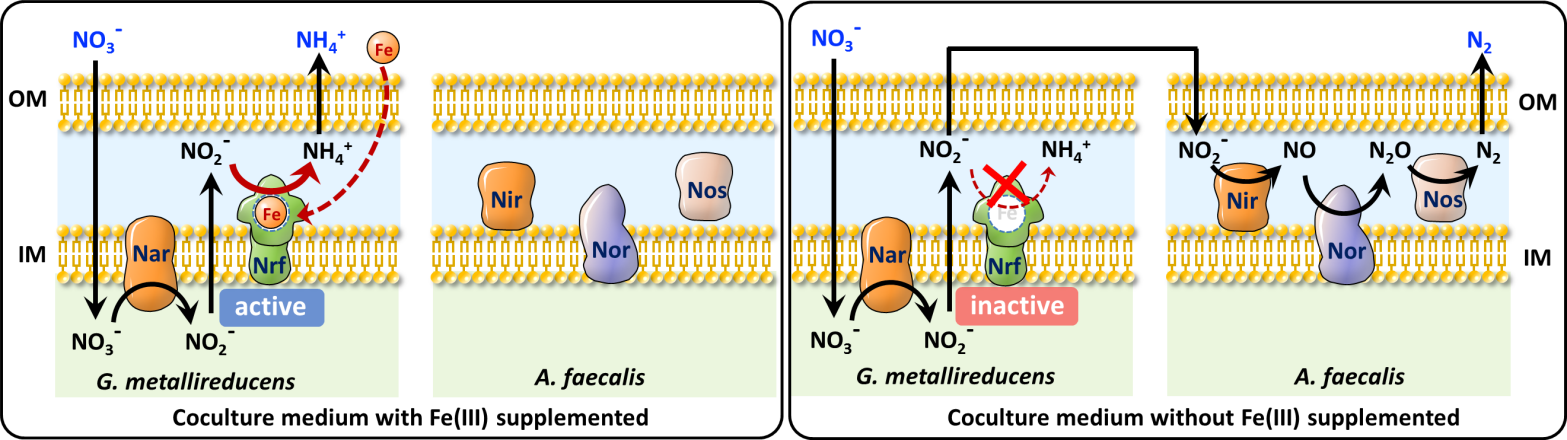
Schematic of how Fe(III) modulates nitrate reduction partitioning in the *G. metallireducens* and *A. faecalis* coculture. Theoretically, *G. metallireducens* reduces nitrate to ammonium, and *A. faecalis* reduces nitrite to nitrogen. After being cocultured in a coculture medium, *G. metallireducens* reduces nitrate to nitrite. In the presence of Fe(III), the nitrite reductase of *G. metallireducens* is active and directly reduces nitrite to ammonium. As a result, a nitrate ammonification coculture forms. However, when Fe(III) is not supplied in the coculture medium, the nitrite reductase of *G. metallireducens* is inactive, and the nitrite cannot be reduced by *G. metallireducens*. Otherwise, nitrite is secreted and transferred to *A. faecalis* for further reduction. Thereafter, a denitrification coculture is established.

To decipher the effects of Fe(III) on the nitrate ammonification by *G. metallireducens*, we measured the catalytic activities of nitrate reductase and nitrite reductase of *G. metallireducens* cell lysates cultured in nitrate medium with different Fe(III) concentrations. As Table S4 and Fig. 4A show, the activity of nitrate reductase in *G. metallireducens* decreased with decreasing Fe(III) culturing concentration, but a moderate nitrate reduction activity of 66 ± 9 nmol min^−1^ mg^−1^ remained when no Fe(III) was provided in the culture medium. Similarly, the nitrite reductase (Nrf) activity also decreased with decreasing Fe(III) concentration. However, the nitrite reductase activity decreased to less than 2 nmol min^−1^ mg^−1^ in the absence of Fe(III) (Fig. 4A). In contrast, the nitrite reductase activity of *A. faecalis* was significant even in the absence of Fe(III) (Table S4). Therefore, the Nrf activity of *G. metallireducens* strictly depends on Fe(III). It appears to be reasonable because the catalytic center of Nrf contains multi-Fe(III) hemes (Fig. 4A) (55). Therefore, we speculate that Fe(III) tunes the nitrate ammonification activity of *G. metallireducens* in the *G. metallireducens* and *A. faecalis* coculture by tuning the Nrf activity. To demonstrate this speculation, we deleted the gene that encoded nitrite reductase in *G. metallireducens* (strain G.m-Δ*nrfA*), cocultured this mutant strain with *A. faecalis,* and controlled the availability of Fe(III) in the coculture medium. As expected, the strain G.m-Δ*nrfA* could not perform DNRA, but it could slightly reduce nitrate and generate a small amount of nitrite in the nitrate medium (Fig. 4B and Fig.S8). This result is not surprising because the strain G.m-Δ*nrfA* still contains nitrate reductase, which can reduce nitrate to nitrite, whereas the generated nitrite is toxic to cells. In contrast, the strain G.m-Δ*nrfA* formed synergistic denitrification with *A. faecalis* in the coculture medium regardless of whether the medium was supplemented with or without Fe(III) (Fig. 4C and D). Therefore, the Nrf activity-dependent nitrate reduction partitioning in the *G. metallireducens* and *A. faecalis* coculture could be deduced from these results (Fig. 5).

### Fe(III) affects nitrate reduction partitioning in urban river water

**Fig 6:**
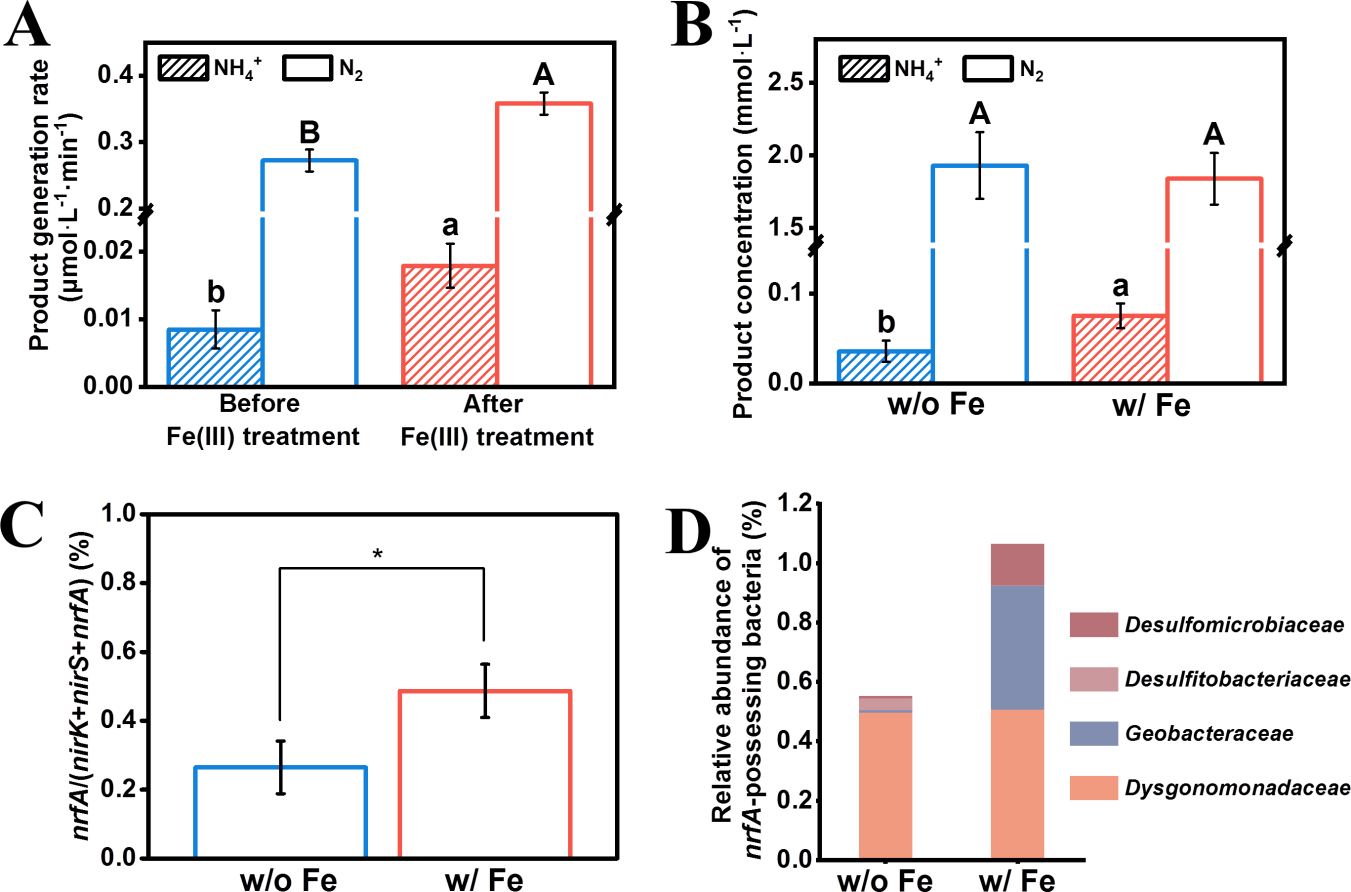
Nitrogen conversion and nitrate-reducing community in urban river water. (**A**) Rates of nitrate ammonification and denitrification in the urban river water microbial community before and after the treatment with Fe(III). (**B**) Final concentrations of ammonium and nitrogen after the nitrate reduction. Significantly different groups are indicated with different letters (LSD test, *P* < 0.05). (**C**) Percentage of *nrfA* to nitrite reductase genes in the microbial community. A two-tailed Student’s t test was performed to identify significant differences among the groups. ^∗^*P* < 0.05, *n* = 3. (**D**) Composition of nitrate ammonifiers in the bacterial community. The water was supplemented with or without Fe(III) (0.3 mmol·L^−1^).

Fe(III)-based cytochrome *c* nitrite reductase has widely been used by nitrate ammonifiers to reduce nitrite to ammonia (29). To identify the possibility that Fe(III) can affect nitrate reduction partitioning in natural environments, we directly examined nitrate reduction partitioning in urban river water contaminated by nitrate after treatment with or without Fe(III). The sampling site is located at Liuhua River (119°15′4.61″E, 26°1′31.36″N). The water contained 4.87 ± 0.03 mmol·L^−1^ nitrate, 0.01 ± 0.00 mmol·L^−1^ nitrite, and 0.06 ± 0.03 mmol·L^−1^ ammonium, whereas the total organic carbon content was only 0.04 ± 0.03 mmol·L^−1^. To stimulate nitrate reduction, 5 mmol·L^−1^ acetate was supplemented as previously reported (6); thereafter, the nitrogen conversion was monitored (Fig. S9). As Fig. 6A shows, the culture mainly performed denitrification due to the C/NO_3_^−^ ratio (ca. 1) being favorable for denitrification. When supplemented with Fe(III), nitrate ammonification in the nitrate reduction immediately increased, which indicates the activation of Nrf. As a consequence, ammonium was finally accumulated (Fig. 6B). Additionally, the presence of Fe(III) ultimately also altered the composition of the microbial community, with increasing nitrate ammonifiers both in the nitrate-reducing community (Fig. 6C) and the entire microbial community (Fig. 6D). Specifically, the microbial community analysis shows that nitrate ammonifiers in urban river water are members of the families *Dysgonomonadaceae*, *Geobacteraceae*, *Desulfitobacteriaceae,* and *Desulfomicrobiaceae*, whereas the Fe(III) treatment increased all nitrate ammonifiers except *Desulfitobacteriaceae*. Therefore, Fe(III) not only acts as a key factor to determine the activity of Nrf but also affects the abundance of nitrate ammonifiers in the nitrate-reducing community, both of which control the nitrate reduction partitioning in the natural nitrate-reducing environment. Notably, the solubility of Fe(III) is governed by pH and organic matter, which, in turn, influences the partitioning of nitrate reduction pathways. Additionally, Fe(III) can act as a competing electron acceptor for nitrate reduction. These interactions drive observed variations in nitrate reduction and highlight the necessity of integrating local geochemical conditions into assessments of nitrate reduction dynamics in natural environments.

### Conclusions

Nitrate reduction partitioning determines the nitrogen conservation in the environment. Various environmental factors have been thought to modulate nitrate reduction partitioning by affecting the structure of the nitrate-reducing community. In this study, we demonstrated that Fe(III) could tune the Nrf activity of the nitrate-reducing community to modulate nitrate reduction partitioning. Specifically, the Nrf activity is Fe(III)-dependent. Therefore, under ferruginous conditions, the Nrf of *G. metallireducens* is active, *G. metallireducens* can reduce nitrate to ammonium when cocultured with *A. faecalis*, and DNRA dominates the nitrogen conversion of the coculture. In contrast, under non-ferruginous conditions, the Nrf of *G. metallireducens* is inactive, and *G. metallireducens* cannot reduce nitrite to ammonium but performs synergistic denitrification with *A. faecalis* via interspecies nitrite transfer. Under these circumstances, nitrate is mainly converted to nitrogen gas in the coculture. Furthermore, we also showed that the addition of Fe(III) promoted nitrate ammonification in nitrate reduction partitioning in urban river water. These results provide a new explanation for nitrate reduction partitioning in nitrate reduction environments and suggest a new key factor that modulates the nitrogen conversion during nitrate dissipation, which is important for understanding nitrogen conservation in aquatic ecosystems.

## SUPPLEMENTAL MATERIAL

Data S1 mbio.02220-25-s0001.xls Differential transcriptome of *G. metalireducens* under coculture and nitrate-medium conditions.

Data S2 mbio.02220-25-s0002.xls Differential transcriptome analysis of *A. faecalis* under coculture and single-culture conditions.

Supplemental material mbio.02220-25-s0003.pdf Fig. S1 to S9, Tables S1 and S2, and captions for Data S1 and S2.

ASM does not own the copyrights to Supplemental Material that may be linked to, or accessed through, an article. The authors have granted ASM a non-exclusive, world-wide license to publish the Supplemental Material files. Please contact the corresponding author directly for reuse.
