# Supplementary material for: Fe(III)-dependent Nrf activity determines nitrate reduction partitioning in nitrate-reducing communities
Source: mBio. 2025 Sep 30;16(11):e02220-25. doi: 10.1128/mbio.02220-25 (PMC12607771; doi:10.1128/mbio.02220-25)
Supplement: Supplemental material — Fig. S1 to S9, Tables S1 and S2, and captions for Data S1 and S2. [file mbio.02220-25-s0003.pdf]

Supplementary material

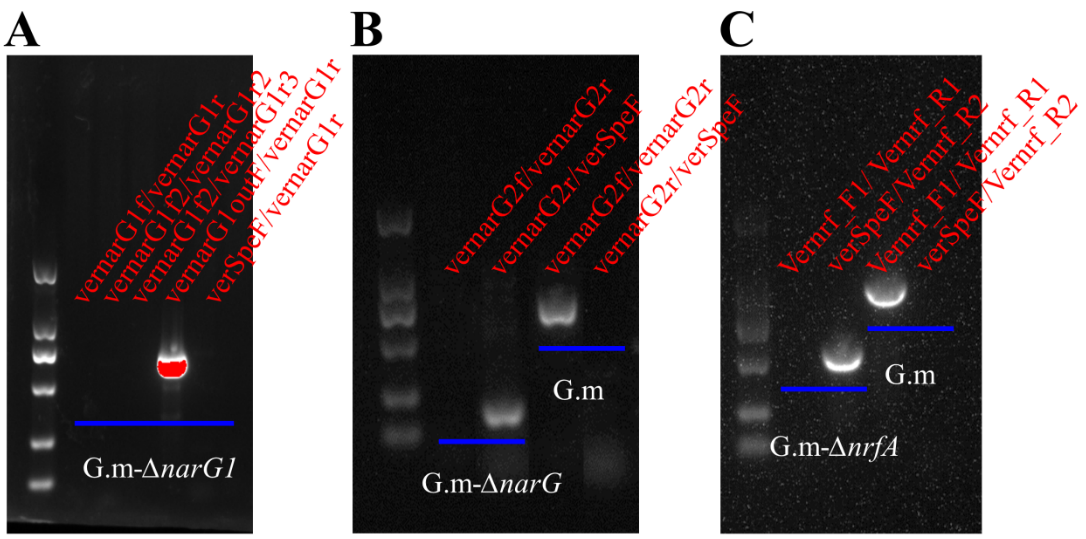

Fig. S1. Verification of mutants of *Geobacter metallireducens* (G.m) used in this study. DNA gels show the PCR results using primers listed in Table S1. Confirmation the construction of (A) G.m-*ΔnarG1*, (B) G.m-*ΔnarG* and (C) G.m-*ΔnrfA* strain. DNA marker DL2000 (Thermo Fisher Scientific, USA) was used as reference.

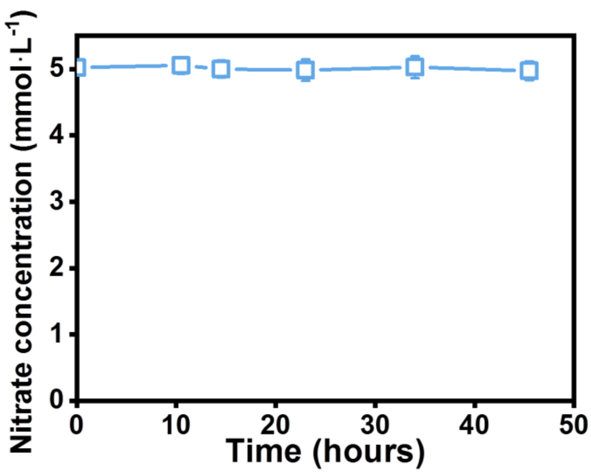

Fig. S2. Nitrate reduction characterization of *Alcaligenes faecalis*.

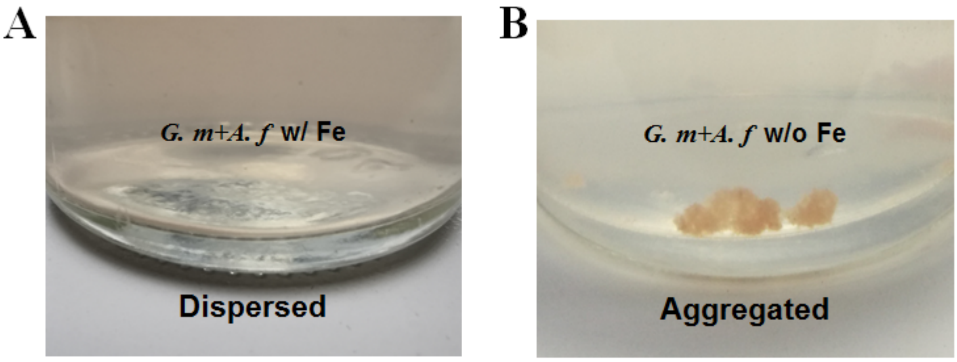

Fig. S3. Appearance *Geobacter metallireducens* and *Alcaligenes faecalis* nitrate-reducing coculture. (A) *G. metallireducens* (G. m) and *A. faecalis* (A. f) coculture growing in coculture medium supplemented with Fe(III). (B) *G. metallireducens* and *A. faecalis* coculture growing in coculture medium without Fe(III)..

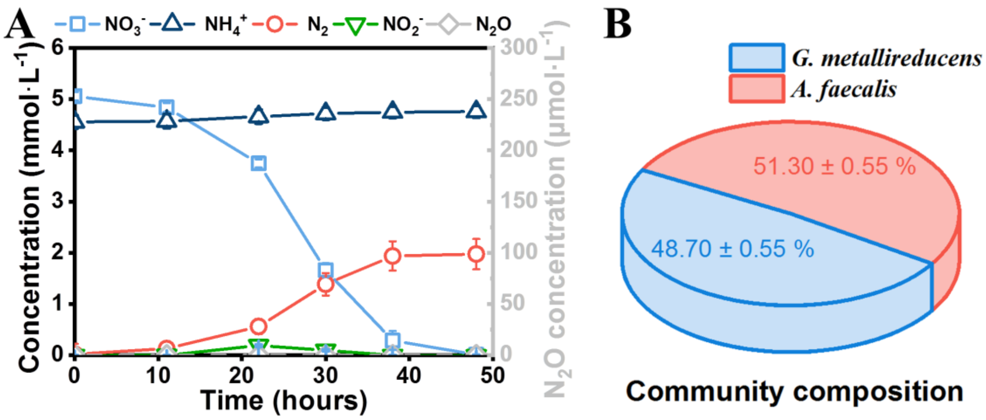

Fig. S4. Characterization of a coculture started with washed *Geobacter metallireducens* and *Alcaligenes faecalis* cells. (A) The nitrogen conversion of the coculture. (B) The proportion of each species in the coculture.

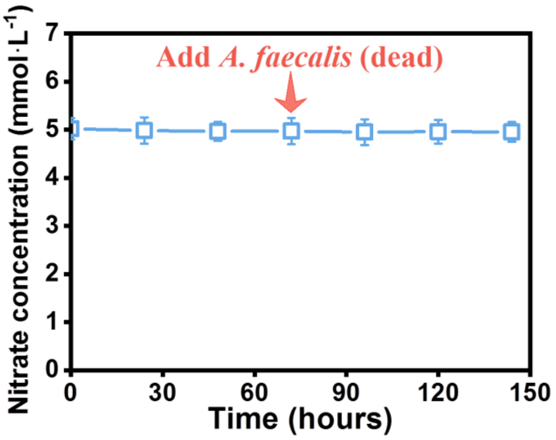

Fig. S5. Nitrate reduction by *Geobacter metallireducens* in a coculture medium. *G.*

metallireducens was washed twice with a coculture medium before inoculation. The addition of dead *Alcaligenes faecalis* was not able to recover the nitrate reduction.

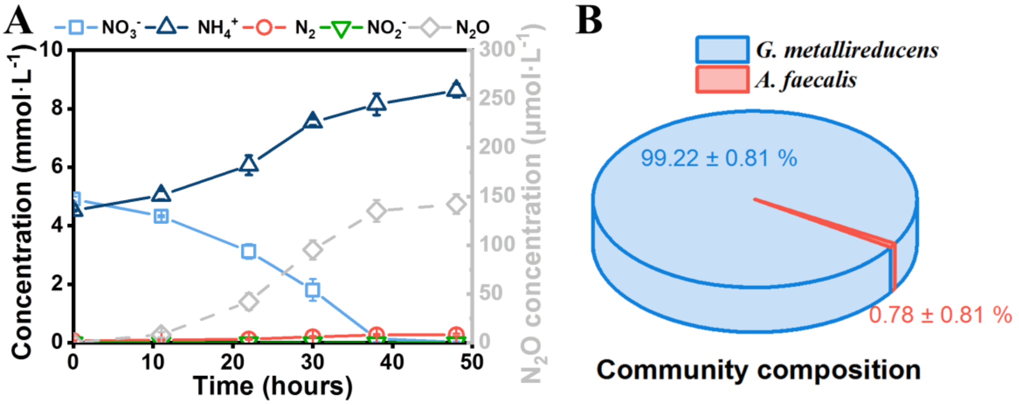

Fig. S6. Characterization of *Geobacter metallireducens* and *Alcaligenes faecalis* coculture with Fe(III) supplement. (A) The nitrogen conversion of *G. metallireducens* and A. *faecalis* coculture. (B) The proportion of each species.

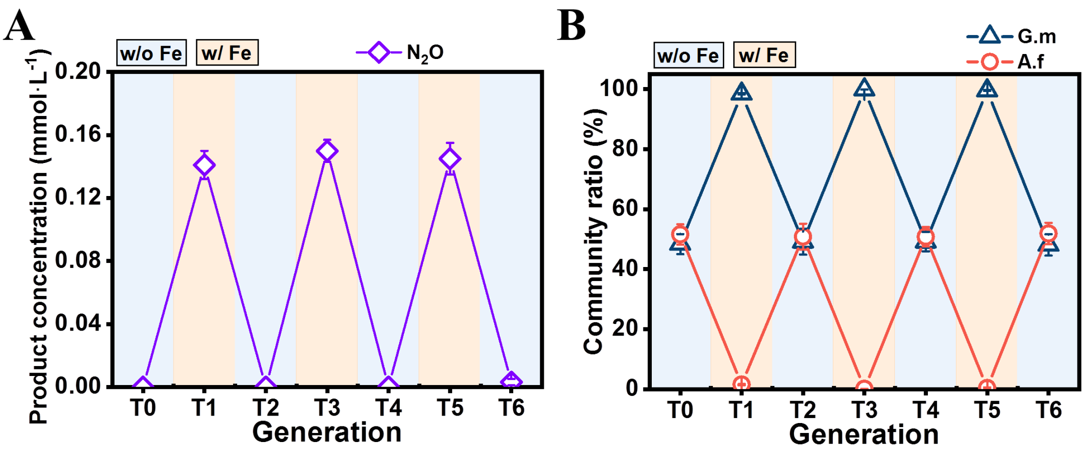

Fig. S7. Characterization of *Geobacter metallireducens* and *Alcaligenes faecalis* coculture treated with Fe(III) intermittently. (A) The production of nitrous oxide. (B) The proportion of *Geobacter metallireducens* (G.m) and *Alcaligenes faecalis* (A.f) in the coculture. The coculture was subcultured six times continuously (from T1 to T6).

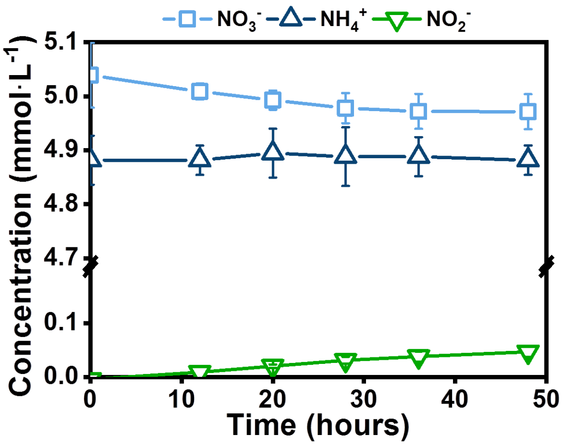

Fig. S8. Nitrogen conversion of *Geobacter metallireducens* mutant strain G.m-Δ*nrfA* (deficient in nitrite reductase) growing in nitrate medium. G.m-Δ*nrfA* could not perform DNRA but slightly reduced nitrate to nitrite.

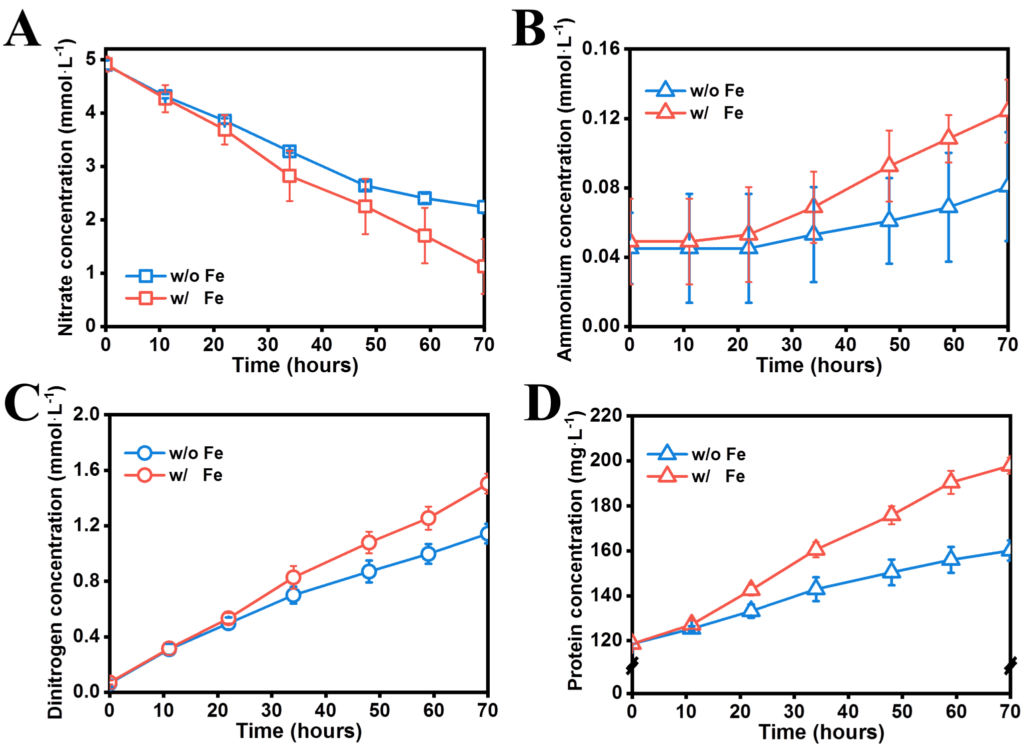

Fig. S9. Nitrogen conversion and biomass increment of nitrate-reducing communities in urban river water. (A) The consumption of nitrate. (B) The production of ammonium. (C) The production of dinitrogen. (D) The increment of microbial protein. The water was supplemented with or without Fe(III).

Table S1. Primers used for mutant construction and qPCR.

Primer name	Amplification/Purpose	Sequence (5' to 3')	Source or reference
GmetFor	pMD19-Gm construction	TTCGGCGTTCCCACCCTCTA	This work
GmetRev	pMD19-Gm construction	CCCCACCTTGAGCATGTCGT	This work
AfF	pMD19-Af construction	CGGTACTGGTTGGCTTGGCTA	This work
AfR	pMD19-Af construction	CAAGCGTCTACGGTCGTTAACC	This work
qGmet_F	G.m quantification	ATGGCCCACATCTTCATCTC	(1)
qGmet_R	G.m quantification	TGCATGTTTTCATCCACGAT	(1)
qAfF	A.f quantification	GGCGATTACCTGCAAAGTTC	This work
qAfR	A.f quantification	GCCAATGGACTGGTTGAAAT	This work
narG1upFor	Gmet_0329 upstream	cggtacccggggatcTCATCATTGAGCCGGACAAGCT	This work
narG1upRev	Gmet_0329 upstream	TCCTCCTCGTTATTGTACTGCCAGCGGTTCCGGTA	This work
narG1dnFor	Gmet_0329 downstream	CCATGGCGGGGAGCGGGCTACGCCCAGTTCAGCTA	This work
narG1dnRev	Gmet_0329 downstream	cgactctagaggatcGTTCGCGCAGTGGTTGCAGAT	This work
narG2upFor	Gmet_1020 upstream	cggtacccggggatcTGGCCAGGATCAGGTCAATGTC	This work
narG2upRev	Gmet_1020 upstream	TCCTCCTCGTTATTGTACGGTAGAATTCCTCCCATCG	This work
narG2dnFor	Gmet_1020 downstream	CCATGGCGGGGAGCGACGAGTGGGTGGAAGTGTACAA	This work
narG2dnRev	Gmet_1020 downstream	cgactctagaggatcCACCCTCCCCGGTACTTCT	This work
spef	*Sp^r-loxp*	CAATAACGAGGAGGA	This work
sper	*Sp^r-loxp*	CGCTCCCCGCCATGG	This work
vernarG1f	Verification the deletion of Gmet_0329	CATCGAGGACAACGAGGAGAT	This work
vernarG1r	Verification the deletion of Gmet_0329	GGCTGGAAGAACATGTTGAGGA	This work
vernarG1f2	Verification the deletion of Gmet_0329	GACGAGGTGACCGAGCTGAT	This work
vernarG1r2	Verification the deletion of Gmet_0329	GGAACGGGAGGTCCGTGTACT	This work
vernarG1r3	Verification the deletion of Gmet_0329	CCACGAGATCCGTCATGTCCA	This work
vernarGoutF	Verification the absence of	GATGACGAAGTAACGAGCCTGAT	This work

verSpeF	spectinomycin cassette in strain G.m-*ΔnarG1*	GAAGGATGTCGCTGCCGACT	This work
vernarG2f	Verification the replacement of Gmet_1020 with *Sp*^{r}::loxp	CGGTCCCTGGAGGAGAAGA	This work
vernarG2r		CTCAAGCTTGTGCACATACACGTA	This work
nrfupFor	Gmet_0295 upstream	cggtacccggggatcCCAGGAAGGGGTACTCATCCA	This work
nrfupRev		TCCTCCTCGTTATTGTACGCCGATCGCCAGGTAGAT	This work
nrfdnFor	Gmet_0294 downstream	CCATGGCGGGGAGCGACCGTGGCCAAACTCTTCGAG	This work
nrfdnRev		cgactctagaggatcCGAGCACAACAGCATCCACAGT	This work
Vernrf_F1	Verification the deletion of Gmet_0294 and Gmet_0295	TGCCACCTCCCCAACGAGAA	This work
Vernrf_R1		GGTGACTGTCTGCGTCCACT	This work
verSpe_F		GAAGGATGTCGCTGCCGACT	This work
Vernrf_R2		CCACCTTCGGGTCAAACGTCA	This work
F1aCu	standards construction and quantification for *nirK*	ATCATGGTSCTGCCGCG	(2)
R3Cu		GCCTCGATCAGRTTGTGGTT	(2)
Cd3aF	standards construction and quantification for *nirS*	GTSAACGTSAAGGARACSGG	(2)
Rc3d		GASTTCGGRTGSGTCTTGA	(2)
nrfAF2aw	standards construction and quantification for *nrfA*	CARTGYCAYGTBGARTA	(2)
nrfAR1		TWNGGCATRTGRCARTC	(2)
515F	16S rRNA sequencing	GTGCCAGCMGCCGCGGTAA	(3)
806R		GGACTACHVGGGTWTCTAAT	(3)

The sequences with yellow background represent 15 bp overlapping sequences required for In-Fusion cloning.

Table S2. Parameters of qPCR.

Aim of qPCR	Primers	Annealing temperature (℃)	Efficiency %	Slope
G.m quantification	qGmet_F/qGmet_R	60	103.4	-3.24
A.f quantification	qAfF/qAfR	60	105.0	-3.21
nirK gene quantification	F1aCu/R3Cu	53	97.4	-3.39
nirS gene quantification	Cd3aF/Rc3d	53	96.4	-3.41
nrfA gene quantification	nrfAF2aw/nrfAR1	50	90.2	-3.58

Table S3. Bacterial strains and plasmids used in this study

Strain or plasmid	Characteristics	Source or reference
Strains		
E. coli		
DH5α	F^-, φ 80d*lacZ* ΔM15, Δ(*lacZYA –argF*) U169, *deoR*, *recA1*, *endA1*, *hsdR17* (rK^-, mK^+), *phoA*, *supE44*, λ^-, *thi-1*, *gyrA96*, *relA1*	Takara
G. metallireducens		
GS15 (ATCC-53774)	Wild type	(4)
G.m-*ΔnarG1*^{Sp}	Δ*Gmet_0329::Sp*^{r}	This work
G.m-*ΔnarG1*	Δ*Gmet_0329*	This work
G.m-*ΔnarG*	Δ *Gmet_0329,* Δ*Gmet_1020*	This work
G.m-*ΔnrfA1*^{Sp}	Δ*Gmet_0294::Sp*^{r}	This work
G.m-*ΔnrfA1*	Δ*Gmet_0294*	This work
G.m-*ΔnrfA*	Δ *Gmet_0294,* Δ*Gmet_0296*	This work
A. faecalis		
DSM30030	Wild type	(5)
Plasmids		
pMD19-T	TA Cloning vector; *Amp*^{r}	Takara
pUC19	In-Fusion Cloning vector; *Amp*^{r}	Takara
pRG5	Spectinomycin resistance gene, *Sp*^{r}	(4)
pCM158	Cre recombinase expression vector; *Km*^{r}	(4)
pMD19-Gm	pMD19-T carrying 500bp sequence of Gmet_2143, *Amp*^{r}	This work
pMD19-Af	pMD19-T carrying 500bp sequence of CPY64_00555- CPY64_00560, *Amp*^{r}	This work
pUC-GMnarG1	pUC19 carrying 500bp upstream and downstream of Gmet_0329 and spectinomycin resistance cassette flanked by *loxP* sites, *Amp*^{r}*, Sp*^{r}	This work
pUC-GMnarG2	pUC19 carrying 500bp upstream and downstream of Gmet_1020 and spectinomycin resistance cassette flanked by *loxP* sites, *Amp*^{r}*, Sp*^{r}	This work
pUC-GMnrfA1	pUC19 carrying 500bp upstream and downstream of Gmet_0294 and spectinomycin resistance cassette flanked by *loxP* sites, *Amp*^{r}*, Sp*^{r}	This work

pUC-GMnrfA2	pUC19 carrying 500bp upstream and downstream of Gmet_0296 and spectinomycin resistance cassette flanked by *loxP* sites, *Amp*^{r}, *Sp*^{r}	This work

Sp^r Spectinomycin resistance; *Amp*^{r}, Ampicillin resistance; *Km*^{r}, Kanamycin resistance.

Table S4. Nitrate and nitrite reductase activities of cell lysates. Cells were grown in coculture medium supplemented with different concentrations of Fe(III).

Strain	Fe(III) addition (μmol·L^{-1})	Reducing rate (nmol min^{-1} mg^{-1})	
		Nitrate	Nitrite
Geobacter metallireducens	5000	195 ± 23	121 ± 11
	1000	122 ± 7	138 ± 8
	250	96 ± 12	88 ± 13
	50	77 ± 8	2 ± 15
	0	66 ± 9	0 ± 7
Alcaligenes faecalis	5000	0 ± 0	70 ± 2
	1000	0 ± 0	44 ± 2
	250	0 ± 0	36 ± 3
	50	0 ± 0	22 ± 2
	0	0 ± 0	27 ± 2

Data S1. (Separate file)
Differential transcriptome of *Geobacter metalireducens* under coculture and nitrate-medium conditions

Data S2. (Separate file)
Differential transcriptome analysis of *Alcaligenes faecalis* under coculture and single-culture conditions.

References

1. **Summers ZM, Fogarty HE, Leang C, Franks AE, Malvankar NS, Lovley DR.** 2010. Direct exchange of electrons within aggregates of an evolved syntrophic coculture of anaerobic bacteria. Science **330**:1413-1415.
2. **Zhang X, Yao C, Zhang B, Tan W, Gong J, Wang GY, Zhao J, Lin X.** 2023. Dynamics of benthic nitrate reduction pathways and associated microbial communities responding to the development of seasonal deoxygenation in a coastal mariculture zone. Environ Sci Technol **57**:15014-15025.
3. **Chen J, Xiao Q, Xu D, Li Z, Chao L, Li X, Liu H, Wang P, Zheng Y, Liu X, Qu H, Bao Y.** 2023. Soil microbial community composition and co-occurrence network responses to mild and severe disturbances in volcanic areas. Sci Total Environ **901**:165889.
4. **Liu X, Zhuo S, Rensing C, Zhou S.** 2018. Syntrophic growth with direct interspecies electron transfer between pili-free *Geobacter* species. ISME J **12**:2142-2151.
5. **Yu L, Yuan Y, Rensing C, Zhou S.** 2018. Combined spectroelectrochemical and proteomic characterizations of bidirectional *Alcaligenes faecalis*-electrode electron transfer. Biosens Bioelectron **106**:21-28.
